# Collaborative Teaching and Learning: A Model for Building Capacity and Partnerships to Address NTDs

**DOI:** 10.1371/journal.pntd.0000939

**Published:** 2011-03-29

**Authors:** Mary Elizabeth Wilson, Albert Icksang Ko, Mitermayer G. Reis

**Affiliations:** 1 Department of Global Health and Population, Harvard School of Public Health, Boston, Massachusetts, United States of America; 2 Yale School of Public Health, Epidemiology of Microbial Disease Division, New Haven, Connecticut, United States of America; 3 Fundação Oswaldo Cruz, Salvador, Bahia, Brazil; Escola Bahiana de Medicina e Saúde Pública and Faculdade de Medicina da Bahia, Federal University of Bahia, Salvador, Bahia, Brazil; René Rachou Research Center, Brazil

Education, training, and a broad understanding of the social, political, economic, and environmental factors underlying poor health and health disparities are key elements in dealing with neglected tropical diseases (NTDs). We have now completed 3 years of the Harvard-Brazil Collaborative Public Health Field Course, and we believe this model is effective in educating students and in building networks for future research, education, and policy. We developed an innovative course curriculum that is multidisciplinary and multi-institutional and aims to prepare students, faculty, and researchers to find new approaches to reducing the burden of NTDs. Four of the five disease examples covered in the course are NTDs: dengue, leishmaniasis, leptospirosis, and schistosomiasis (the fifth is HIV/AIDS).

Based on interactions with students in the classroom, we concluded that there was a need for students to spend time in settings where these diseases persist to better understand that interventions must draw upon knowledge and experience from multiple sectors and disciplines. We wanted students to break out of the traditional academic environment and engage in solving immediate real-world problems. The placement of a field office of the David Rockefeller Center for Latin American Studies at Harvard in São Paulo made it feasible to support educational activities in Brazil.

The course is offered in January each year and is taught in Brazil by Brazilian and Harvard faculty over a period of almost 3 weeks. Fifteen Harvard students (graduate students at the Harvard School of Public Health) and 15 Brazilian students from many institutions and areas of Brazil are selected based on applications and interviews. Selection is based in part on the expected benefit to the student in a career in a health-related field.

The students are an exceptional group. They come from many disciplines (e.g., anthropology, law, engineering, health policy, biomedicine, nursing, veterinary medicine, nutrition, statistical methods, immunology, ecology, economics, environmental sciences, and others) and bring rich and varied background experiences to the class. Many of the Harvard students are from non-US countries, including Ghana, Singapore, Japan, Korea, Scotland, and Nigeria, among others in past years. Students from Brazil are required to speak English (lectures and class discussion are in English) and are selected from academic and public health institutions, including the national Field Epidemiology Training Program (FETP), and have the potential to develop into future NTD leaders. Most of the Harvard students speak one or more non-English languages, but few are fluent in Portuguese.

The structure of the course involves lectures, classroom discussions, and visits to field sites, clinical settings, and laboratories during the first week of the course. Lectures introduce students to the health care system in Brazil, and cover human rights, demographic methods, and working with data, as well as content about NTD-specific issues that affect the region. In Salvador, Brazil (Bahia), where we held the course the last two years, the students (in small groups) visit a favela community, accompanied by local research teams, and talk with residents and community leaders who are participating in long-term longitudinal studies on leptospirosis and other emerging urban health problems (see Figure 1). They also spend time with the municipal dengue control team in the field, going into the communities to visit homes to look for breeding sites with immature forms of *Aedes aegpyti*, the main vector for the dengue fever virus, a major and growing infectious disease menace in Salvador and elsewhere. They learn about some of the diagnosis and control efforts, e.g., by observing the techniques for identifying schistosome ova in fecal specimens and observing the laboratory approaches to identify mosquito larvae. The students also visit the state infectious disease hospital, where they talk with patients with HIV and other diseases. In the hospital they also see problems rarely seen in the US, such as tetanus and neurologic complications of schistosomiasis.

**Figure 1:**
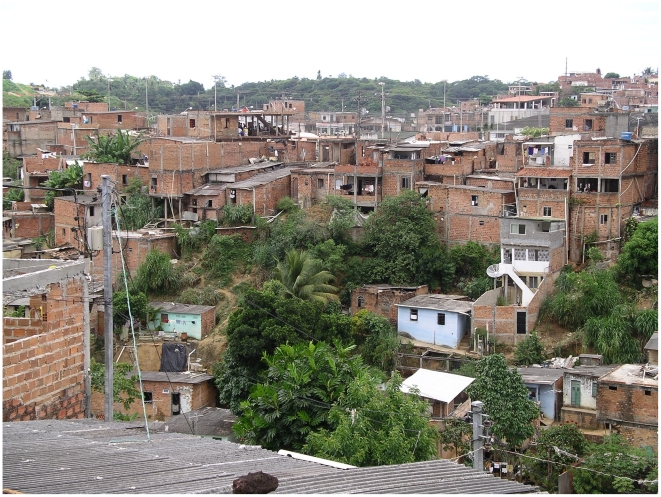
Favela community site for the course in the city of Salvador, Brazil, where leptospirosis is an important health problem. Photo taken by Albert Ko.

The five diseases selected for the course are used as a means to help students understand and research complex, multi-faceted health problems. Societal factors, such as socioeconomic status, behavior, and living conditions, strongly contribute to risk for NTDs. Broad knowledge across multiple disciplines is needed to develop effective approaches for the control of HIV/AIDS and NTDs. Three of these diseases—dengue, HIV/AIDS, and leptospirosis are a greater problem in urban areas, and two (leishmaniasis and schistosomiasis) are concentrated in rural areas (though urbanization of the latter two is being observed in some areas) (see Figure 2). Table 1 shows the number of cases and deaths from these diseases reported to the National Surveillance System in Brazil. The range of diseases allows learning about diseases with different modes of transmission (e.g., vector-borne, water-associated, person-to-person) and the different classes of organisms (viruses, bacteria, helminths, protozoa) that cause them.

**Figure 2:**
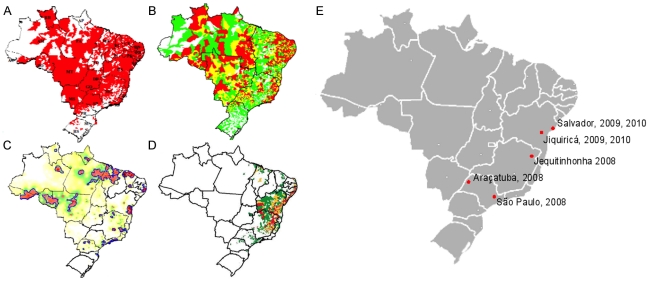
Disease incidence and vector distribution and location of Harvard-Brazil course, 2008–2010. (A) Distribution of *Aedes aegypti* infestation according to municipality, 2001 [1]. (B) Dengue incidence rate by municipality, 2008 (red, yellow, and green are high, medium, and low risk municipalities, respectively) [2]. (C) Kernel distribution of reported cases of tegumentary leishmaniasis, 2004–2006 (red, blue, green, and yellow represent high to low risk areas) [3]. (D) Prevalence of schistosomiasis according to municipality, 2006 (red, yellow, and green are high, medium, and low prevalence municipalities, respectively) [4]. (E) Location of Harvard-Brazil Collaborative Course sites, 2008–2010.

**Table 1:** Cases and deaths due of selected NTDs that are reported to the National Surveillance System in Brazil.

NTD	Year	No. Reported Cases	No. Reported Deaths
Schistosomiasis	2007	113,712 [5]	608 [6]
Dengue	2008	585,769 [7]	249 [8]
Tegumentary leishmaniasis	2009	21,824 [9]	—
Visceral leishmaniasis	2006	3,651 [10]	262 [11]
Leptospirosis	2008	3,542 [12]	334 [13]

The reported cases and deaths underestimate the actual numbers because of lack of laboratory confirmation.

All of the students and some of the faculty spend at least 2–3 days in a rural area, several hours drive from Salvador, where leishmaniasis and schistosomiasis remain endemic. Students talk with local researchers, public health workers, and local residents—and importantly, they can observe how people live and use the local rivers for activities of daily life, including bathing and laundry. They can also observe untreated sewage flowing into the streams.

During the second week of the course, students work together in problem-oriented, multidisciplinary teams of six (three Brazilian and three Harvard students), each supported by assigned faculty. The students are charged with identifying a question that needs to be answered in order to control or reduce the burden of the disease assigned to that group. The team develops a practical research proposal that would help answer the question. The students draw on data from electronic databases, published literature, and discussions with local researchers, public health workers, and residents. By design, each team typically has students with diverse backgrounds, and they are asked to draw on the experiences and training of all team members in developing their proposals. Teams present their preliminary proposal to the local community to receive feedback before refining it and presenting it to the entire class and faculty at the end of the course. We ask that the proposals be feasible, tailored to the needs of the local community, fundable, and designed to inform policy or public health decisions.

The final presentations reflect collaborative efforts and group learning. When each team presents their proposal to the entire class, all members of the team participate in the presentation, respond to questions, or both. Ample time is available for discussion; responses reflect a remarkable mastery of content by the students in a relatively short period of time.

The proposals developed by the students are varied, creative, and reflect the wide range of training and experiences among the participants. For example, proposals developed in 2010 included:

- Dry Toilets: The Green Solution for Schistosomiasis Control
- Evaluation of Water Tank Covers to Control Dengue in Salvador
- Garbage and Leptospirosis: A Study of Knowledge, Attitudes, and Practices in Pau da Lima and Assessment of Community-based Trash Collection
- Testing All Mothers: Issues Impacting Pregnant Women's Completion of HIV/AIDS Testing in Salvador, Bahia
- Environmental Mnagement and Sandfly Vector Control in the Prevention of Leishmaniasis

Throughout the course, students from Brazil and Harvard live, travel, work, and socialize together. They experiment with local food and enjoy music and dancing events. Students learn to accommodate to different learning styles and approaches to problem solving. Partners are essential, and we have many. The Oswaldo Cruz Foundation (Fiocruz) in Salvador has been our primary partner for the last 2 years, and lectures and closing sessions are held at that facility. The David Rockefeller Center for Latin American Studies at Harvard (field office in São Paulo) and their staff have provided key support for the development and completion of the course from the outset. We typically have four professors from Harvard who teach in the course, with two or three present for the entire course. In 2010, in addition to faculty from Fiocruz in Salvador, we had faculty from the University of São Paulo, the Santa Casa Medical School in São Paulo, and the Federal University of Bahia. Other participant partners included the Hospital Couto Maia, the cities of Jiquirica and Ubaira in Bahia, and Centro de Controle de Zoonoses and Unidade Municipal de Saude de Sao Marcos. We also received support from the Conselho Nacional de Pesquisas (CNPq), the Fundacao de Amparo a Pesquisa do Estado da Bahia (FAPESB), and the Harvard Institute for Global Health (HIGH).

Harvard students have to pay for most of the cost of travel and accommodations. The Harvard School of Public Health pays for 25% of coach airfare to Brazil and a small weekly stipend. Each student is individually recognized and awarded a certificate on the last day of the course; Harvard students receive academic credit for the course.

The course has evolved over the 3 years, and we expect it to continue to change. We have added a session (with computers) to help students learn how to access valuable databases. Because many of the students have little experience in study design, we will add a practical session on this topic next year. We have reduced the number of lectures and simplified the number of site visits and field sites. We have moved all of the lectures to the first week of the course and have started the team meetings earlier in the course.

We believe that we have met most of the learning objectives, which were to:

- Gain an understanding of the socioeconomic, environmental, political, cultural, and biological factors associated with NTDs
- Engage local residents, researchers, and field workers to learn about their needs, obstacles, and priorities
- Define research questions and develop research proposals to advance control of these diseases
- Build a multi-disciplinary and multi-country network of friends and collaborators to facilitate future research and education
- Gain experience in multi-disciplinary, team-based problem solving
- Begin to understand another culture through shared experiences—academic, social, and cultural—which is the first step to building successful public health and research initiatives and collaborations

## Outcomes

Since the first course in 2008, we have seen at least seven Harvard students complete master's thesis projects that drew on their experiences in Brazil (see Box 1); two Harvard students—one of whom had a Fulbright Fellowship—are currently doing research in Brazil for their doctoral theses; one Brazilian student has completed a Master's of Public Health degree at Harvard, and another is doing research supported by the US National Institutes of Health. One of the student team proposals from 2009 was included as part of a larger project funded in Salvador. Course faculty from Brazil have presented lectures and participated in a symposium at Harvard, and one has been appointed as a visiting scientist at Harvard. Informally, many of the students and faculty have continued to exchange ideas. The course has also served as a model that has inspired other collaborative initiatives involving faculty and students from Brazil and from Harvard in other disciplines.

**Box 1. Master's Thesis Work following Harvard-Brazil Course**

Controlling American Visceral Leishmaniasis in São Paulo

Implications of the Emergence of Dengue Virus Serotype 4 in Brazil

Chemoattractant Sticky Traps in Dengue Vector Suppression

Combating Malarial Associated Anemia in Pregnancy: Is Empirical Oral Iron Therapy an Option?

Schistosomiasis and Sanitation: Social Causes of the Persistence of a Preventable Disease in Brazil and Opportunities for Reform

Analysis of the Epidemiology of Dengue Fever and Control Programs in Brazil

Gender Dynamics and Contraceptive Use Decision-Making within Women's Intimate Partner Relationships in Metropolitan São Paulo, Brazil*

* The student who did this work returned to Brazil after the course to collect data. The thesis was awarded the prize for best master's thesis of the graduating 2009 cohort of the Department of Global Health and Population, Harvard School of Public Health.

## The Future

Key elements of the course include diversity in countries, cultures, and disciplines; inclusion of laboratory research and community-based research; academic and public institutions; urban and rural populations; and collaboration among students and faculty in shared learning and in building networks. We aim to establish and reinforce relationships among faculty, students, and partner institutions to generate opportunities for future research, teaching, and field work that will improve the health of populations globally. The course is preparing future leaders with a multi-dimensional vision of how to address NTDs. We expect that the benefits from the course will continue to be realized years and decades from now and consider this an excellent investment to improve global health—and, especially, to help reduce the burden of NTDs.
