# Correction: Collaborative Teaching and Learning: A Model for Building Capacity and Partnerships to Address NTDs

**DOI:** 10.1371/annotation/d33a8e45-4a41-49c7-81b1-1355a4c28a81

**Published:** 2011-08-12

**Authors:** Mary Elizabeth Wilson, Albert Icksang Ko, Mitermayer G. Reis

Several authors were not included in the author byline. Please see the correct list of authors, affiliations, and citation here:

Mary Elizabeth Wilson1*, Felipe Fregni2, Maria Amelia de S.M.Veras3, Jason Dyett4, Amie Shei5, Guilherme Sousa Ribeiro6, John David7, Marcia C. Castro1, Aluisio Cotrim Segurado8, Albert Icksang Ko9, Mitermayer G. Reis10

1 Department of Global Health and Population, Harvard School of Public Health, Boston, Massachusetts, United States of America, 2 Harvard Medical School; Spaulding Rehabilitation Hospital and Massachusetts General Hospital, Boston, Massachusetts, United States of America, 3 Department of Social Medicine, School of Medical Sciences, Santa Casa do São Paulo, São Paulo, Brazil, 4 Brazil Office, David Rockefeller Center for Latin American Studies at Harvard, São Paulo, Brazil, 5 Harvard University Ph.D. Program in Health Policy, Cambridge, Massachusetts, United States of America, 6 Instituto de Saude Coletva, Universidade Federal da Bahia and Fundacao Oswaldo Cruz, Salvador, Bahia, Brazil, 7 Department of Immunology and Infectious Diseases, Harvard School of Public Health, Boston, Massachusetts, United States of America, 8 Department of Infectious Diseases, School of Medicine, University of São Paulo, São Paulo, Brazil 9 Yale School of Public Health, Epidemiology of Microbial Disease Division, New Haven, Connecticut, United States of America, 10 Fundação Oswaldo Cruz, Salvador, Bahia, Brazil; Escola Bahiana de Medicina e Saúde Pública and Faculdade de Medicina da Bahia, Federal University of Bahia, Salvador, Bahia, Brazil

Wilson ME, Fregni F, Veras MASM, Dyett J, Shei A (2011) Collaborative Teaching and Learning: A Model for Building Capacity and Partnerships to Address NTDs. PLoS Negl Trop Dis 5(3): e939. doi:10.1371/journal.pntd.0000939

